# Indoxyl Sulfate-Induced Valve Endothelial Cell Endothelial-to-Mesenchymal Transition and Calcification in an Integrin-Linked Kinase-Dependent Manner

**DOI:** 10.3390/cells13060481

**Published:** 2024-03-08

**Authors:** Maria Delgado-Marin, Sandra Sánchez-Esteban, Alberto Cook-Calvete, Sara Jorquera-Ortega, Carlos Zaragoza, Marta Saura

**Affiliations:** 1 Unidad de Fisiología, Departamento de Biología de Sistemas, Facultad de Medicina, IRYCIS, Universidad de Alcalá, 28871 Alcalá de Henares, Spain; maria.delgado@uah.es (M.D.-M.); sandra.sancheze@uah.es (S.S.-E.); alberto.cook@uah.es (A.C.-C.); sara.jorquera@uah.es (S.J.-O.); 2 Cardiovascular Research University Francisco de Vitoria and Hospital Ramon y Cajal, IRYCIS, 28034 Madrid, Spain; c.zaragoza.prof@ufv.es; 3 Centro de Investigación Biomédica en Red de Enfermedades Cardiovasculares (CIBERCV), Instituto de Salud Carlos III, 28029 Madrid, Spain

**Keywords:** valve endothelial cells, calcific valve disease, chronic kidney disease, indoxyl sulfate, cell transdifferentiation, integrin-linked kinase

## Abstract

Calcific Aortic Valve Disease (CAVD) is a significant concern for cardiovascular health and is closely associated with chronic kidney disease (CKD). Aortic valve endothelial cells (VECs) play a significant role in the onset and progression of CAVD. Previous research has suggested that uremic toxins, particularly indoxyl sulfate (IS), induce vascular calcification and endothelial dysfunction, but the effect of IS on valve endothelial cells (VECs) and its contribution to CAVD is unclear. Our results show that IS reduced human VEC viability and increased pro-calcific markers RUNX2 and alkaline phosphatase (ALP) expression. Additionally, IS-exposed VECs cultured in pro-osteogenic media showed increased calcification. Mechanistically, IS induced endothelial-to-mesenchymal transition (EndMT), evidenced by the loss of endothelial markers and increased expression of mesenchymal markers. IS triggered VEC inflammation, as revealed by NF-kB activation, and decreased integrin-linked kinase (ILK) expression. ILK overexpression reversed the loss of endothelial phenotype and RUNX2, emphasizing its relevance in the pathogenesis of CAVD in CKD. Conversely, a lower dose of IS intensified some of the effects in EndMT caused by silencing ILK. These findings imply that IS affects valve endothelium directly, contributing to CAVD by inducing EndMT and calcification, with ILK acting as a crucial modulator.

## 1. Introduction

Calcific Aortic Valve Disease (CAVD) stands as the most prevalent valvopathy in high-income countries, having escalated to become the third leading cause of cardiovascular disease. In 2019, CAVD was responsible for over 120,000 deaths worldwide, mainly affecting people over 65 years old [1]. Due to the steady increase in the aging population, the incidence and prevalence of CAVD is expected to rise in the coming years [2].

Pathologically, CAVD is a chronic disease characterized by the thickening of the aortic valve due to the accumulation of calcium deposits, leading to a significant narrowing of the valve orifice [3,4]. The disease progression is steady and rapid, and although it may initially be asymptomatic, advanced stages of CAVD manifest as heart failure and even premature death [3,5]. At this stage, the only viable treatment option is the surgical replacement of the valve [6]. Besides aging, chronic kidney disease (CKD) is a major risk factor of CAVD associated with earlier development, rapid disease progression, and higher mortality rates than those with similar severity of aortic stenosis but normal renal function [7,8,9,10]. Regardless of this well-known correlation between CAVD and CKD, the molecular mechanisms promoting valve calcification in patients with chronic kidney disease have not been completely elucidated.

Aortic valve endothelial cells (VECs) and aortic valve interstitial cells (VICs) are the main cell types that comprise the aortic valve structure. Nowadays, CAVD is considered a highly active pathological process in which both cell types play a critical role. This process can be divided into two different phases: the initiation and propagation phase. The initiation phase is characterized by endothelial dysfunction, inflammation, and lipid accumulation, highlighting the importance of the integrity of the endothelial barrier at the onset of the disease. The activation of the interstitial cells characterizes the propagation phase and represents the actual calcification phase [5]. Recently, several studies have suggested that valve endothelium can directly contribute to calcification through the endothelial-to-mesenchymal transition (EndMT). During this process, the valve endothelial cells (VECs) lose their specific endothelial phenotype and markers (CD31 and VE-Cadherin) and acquire mesenchymal markers (α-smooth muscle actin and transgelin/SM22α). Thus, transdifferentiated VECs can migrate into the valve interstitium, where they behave as endothelium-derived VICs promoting calcium formation [11]. Indeed, our group has also demonstrated the osteogenic transdifferentiation of VECs via EndMT and has linked this process to reduced levels of integrin-linked kinase (ILK), a serine/threonine kinase crucial to maintaining endothelial integrity in human disease [12,13].

Indoxyl sulfate (IS) is a protein-bound uremic toxin produced through the gut microbiota’s conversion of dietary tryptophan into indole. After absorption, indole reaches the liver, transforming into IS through oxidation and sulfation. While usually cleared through renal tubular secretion, in chronic kidney disease (CKD) patients IS accumulates in blood and tissues, primarily bound to plasma proteins [14]. IS is essential in connecting renal and cardiovascular diseases, directly affecting the endothelium and compromising its functions [15,16,17,18]. IS has been strongly associated with vascular calcification in human pathology [19]. In vitro, IS induces oxidative stress and promotes senescence and calcification in vascular smooth muscle cells [20,21,22,23]. Regarding valvular calcific disease, Candellier et al. have recently described the role of IS in the osteogenic differentiation of VICs in an AhR-NF-κB-dependent manner [24]. Moreover, the administration of IS to hypertensive rats accelerates the aortic calcification [25].

Nevertheless, the potential implications of this toxin on valvular endothelial cells, which play a significant role in the early stages of CAVD, remain largely unknown. Hence, it is imperative to elucidate the effects triggered by indoxyl sulfate in valvular endothelium to clarify the molecular processes occurring in the earliest stages of CAVD in CKD. 

## 2. Materials and Methods

### 2.1. Human Valve Endothelial Cell (hVEC) Isolation

Non-calcified human aortic valves were obtained as surgical residue from patients undergoing valve replacement surgery at the Cardiac Surgery department of the Ramón y Cajal Hospital after obtaining informed consent from the patients. The human samples were obtained under the Declaration of Helsinki (as revised in 2013). The study protocol was approved by the Ethics Board of Hospital Universitario Ramón y Cajal (2018/356).

Valves were kept in DMEM/F12 culture medium supplemented with 10% Fetal Bovine Serum (FBS) from Gibco (Waltham, MA, USA) and 0.05 mg/mL Penicillin/Streptavidin (Lonza, Basel, Switzerland), as described in [12]. Briefly, the valve leaflets were delicately sectioned into 2 mm fragments and cultured in a dish coated with Matrigel (Corning, Madrid, Spain). The valve explants were grown in Endothelial Basal Medium-2 (EBM-2) supplemented with an EGM-2kit (CC4176, Lonza, Basel, Switzerland) for two weeks. The explants were extracted, and the endothelial cells were dissociated from the Matrigel using a 2 U/mL Dispase II solution (Roche, Madrid, Spain) at 37 °C for 1 h. The resultant cell suspension underwent centrifugation at 900 rpm for 5 min, and the resulting cell pellet was seeded onto a gelatin-coated plate. These cells were subjected to stringent characterization techniques such as confocal microscopy, demonstrating that 97.7% of the primary cell culture expressed CD31 while displaying a negative response for αSMA, conclusively confirming the endothelial phenotype. 

Cells were cultured in a controlled and humidified CO_2_ environment and cells from passages 2 to 5 were utilized in subsequent experiments, ensuring the reliability and integrity of the research.

### 2.2. Cell Treatment

The human valve endothelial cells (hVECs) were exposed to indoxyl sulfate (Sigma Aldrich, Burlington, MA, USA) at a concentration of 250 μM or 100 μM for 3, 5 and 7 days. The treatment was replenished every 2–3 days, ensuring sustained exposure until the completion of the experiment. 

### 2.3. MTT Cell Viability Assay 

Human VECs were cultured in 24-well plates with 1000 cells per well in a complete medium. Following exposure to IS for 3, 5, and 7 days at concentrations of 0 (control), 100 μL and 200 μL, 50 μL of MTT solution (5 mg/mL) was introduced to each well in 500 μL of the medium. Then, the plates were incubated for 3.5 h at 37˚ C. Subsequently, the supernatant was aspirated, and DMSO (Sigma Aldrich, Burlington, MA, USA) was employed to dissolve the formazan crystals. The absorbance was measured at a test wavelength of 570 nm [26]. 

### 2.4. Confocal Microscopy 

Cover slips containing human vascular endothelial cells (hVECs) were cultured and then exposed to primary antibodies overnight at 4 °C. Following PBS washing, the coverslips were treated with either Alexa-488- or Alexa-647-conjugated secondary antibodies for 1 h at room temperature. Hoechst dye was utilized to stain the cell nuclei. Image acquisition for data analysis was performed using a Leica TCS SP5 confocal microscope available at the UAH-NANBIOSIS-CIBER-BNN facility. Negative controls were established by excluding primary antibodies. Fluorescent imaging was conducted at a magnification of ×60, with at least five fields analyzed per experimental condition.

### 2.5. RT-qPCR 

Total RNA from hVECs was obtained using the TRIzol reagent from Invitrogen Corporation (Carlsbad, CA, USA), following the manufacturer’s instructions. Next, we synthesized first-strand cDNA using 2 μg of total RNA in a 20 μL reaction volume with the High-Capacity cDNA reverse transcription kit. The qPCR analysis was conducted using the SYBR Select Master Mix, both sourced from Life Technologies (Carlsbad, CA, USA). The primer sequences employed in this study can be found in Table S2.

### 2.6. Immunoblot

Protein lysates were processed according to established protocols [12]. Briefly, tissue samples were homogenized in a protein lysis buffer of Novagen cytobuster protein extraction reagent (EMD chemicals, San Diego, CA, USA), supplemented with Complete Mini and Phospho-stop reagents (Roche). A total of 15 μg of protein was separated using 10% SDS-polyacrylamide gel electrophoresis and subsequently transferred onto PVDF membranes (Bio-Rad, Hercules, CA, USA). 

Membranes underwent blocking for one hour in a 5% BSA/TTBS buffer, followed by overnight incubation with specific antibodies at 4 °C. After several washes, the membranes were exposed to an appropriate secondary antibody. Immunoreactive bands were visualized using the Pierce TM ECL Western Blotting Substrate, following the manufacturer’s guidelines (Pierce, Waltham, MA, USA).

### 2.7. Osteogenic Differentiation Assay

Human VECs were cultured for 15 days in a pro-osteogenic medium comprising DMEM supplemented with 10% FBS, 0.05 mg/mL penicillin/streptomycin, and 2.5 μg/mL amphotericin, along with 10 nmol/L beta-glycerophosphate, 50 μmol/L ascorbic acid, and 10 μmol/L dexamethasone to simulate an osteogenic environment. Control cultures were maintained in DMEM with antibiotics and 10% FBS. 

Following the experimental procedures, cells underwent PBS washing and were fixed using 4% paraformaldehyde for 15 min. Subsequently, the cells were rinsed with PBS. Mineralization assessment was performed through staining with 0.02 mg/L of Alizarin Red S 40 nM (Sigma-Aldrich, Sal Luis, MO, USA). 

The area displaying positive Alizarin Red S staining was standardized, based on the cell count, for accurate quantification. At least four images were taken for every experimental condition using NIS element D3.2 Nikon software (Nikon, Tokyo, Japan) and quantified with ImageJ 2.0.0 software (NIH, Bethesda, MD, USA).

### 2.8. Statistical Analysis

The results are presented as means and the corresponding standard deviations (SDs). Statistical analyses were performed using GraphPad Prism 10.2.1 software (GraphPad Software Inc., San Diego, CA, USA). Each experimental condition was duplicated within each experiment, and each experiment was repeated at least three times. Depending on the experimental design, the student’s *t*-test or one-way ANOVA was employed to determine statistical significance between groups. The normal distribution of variables was confirmed using the Shapiro–Wilk test. Unless otherwise specified, differences between groups were considered significant at *p*-values less than 0.01 (* *p* < 0.01; ** *p* < 0.001; *** *p* < 0.0001).

## 3. Results

### 3.1. Indoxyl Sulfate (IS) Induces Valve Endothelial Cell (VEC) Calcification

**Figure 1:**
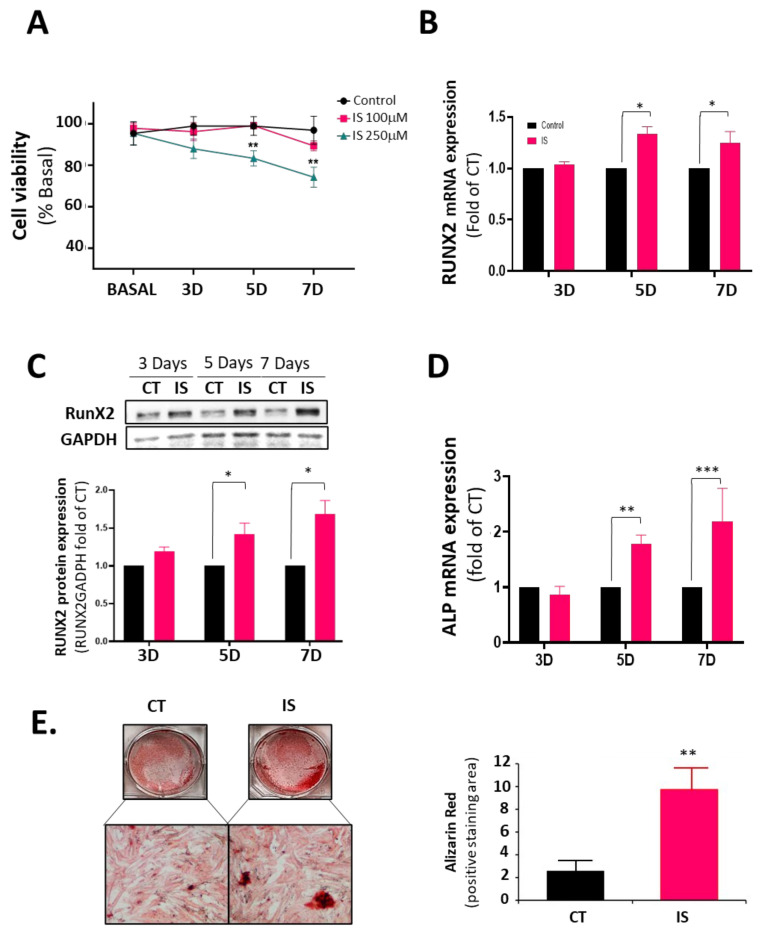
Indoxyl sulfate (IS) induces VEC osteogenic changes. Human valve endothelial cells (VEC) were treated with IS or PBS for 3, 5, and 7 days and harvested for cell viability, mRNA expression, and protein expression studies. (**A**) VECs were treated with IS (250 μM and 100 μM) or PBS and studied for cell viability using an MTT assay. Assays were performed in triplicates (*** *p* < 0.0001; *n* = 3); (**B**) RT-qPCR of CT- and IS-treated hVEC extracts showing mRNA expression of RUNX2 (* *p* < 0.01; *n* = 4); (**C**) Western blot analysis and quantification of RUNX2 in hVECs. Experiments were performed in duplicate (* *p* < 0.01; *n* = 3); (**D**) RT-qPCR of CT- and IS-treated hVEC extracts showing mRNA expression of alkaline phosphatase (ALP) (** *p* < 0.001; *** *p* < 0.0001; *n* = 3); (**E**) hVECs cultured with pro-osteogenic medium for 15 days were treated with IS (250 μM) or with PBS. Scale bar = 100 μm. Right panel: Quantification of alizarin-red positive area. *n* = 6. ** *p* < 0.001 vs. CT.

To test the effect of indoxyl sulfate on human valve endothelial cells, we first conducted a viability assay for 3, 5, and 7 days using two IS concentrations: 250 μM and 100 μM. The 250 μM dose is a high dose, similar to the average IS concentration found in patients with CKD at stage 5 [27,28] (Figure 1A). As expected, 250 μM IS induced hVEC toxicity at five and seven days of treatment, whereas 100 μM did not significantly reduce cell viability. Unless otherwise stated, we used the pathological concentration of 250 μM for the rest of the study. 

Next, we studied the mRNA expression of RUNX2 in response to IS. RUNX2 is a key transcription factor associated with early osteoblastic differentiation in VECs and VICs [29,30]. IS induced a 1.5-fold increase in *RUNX2* mRNA after five days (Figure 1B). RUNX2 protein expression paralleled this result (Figure 1C). In addition, alkaline phosphatase mRNA expression also increased at the same time points (Figure 1D). The above results suggested that IS treatment activates VECs to express molecules critical in valve calcification. Accordingly, VECs cultured in osteogenic media and treated with IS for seven days showed a 10-fold increase in calcification (Figure 1E).

### 3.2. IS Induces VEC Transdifferentiation

**Figure 2:**
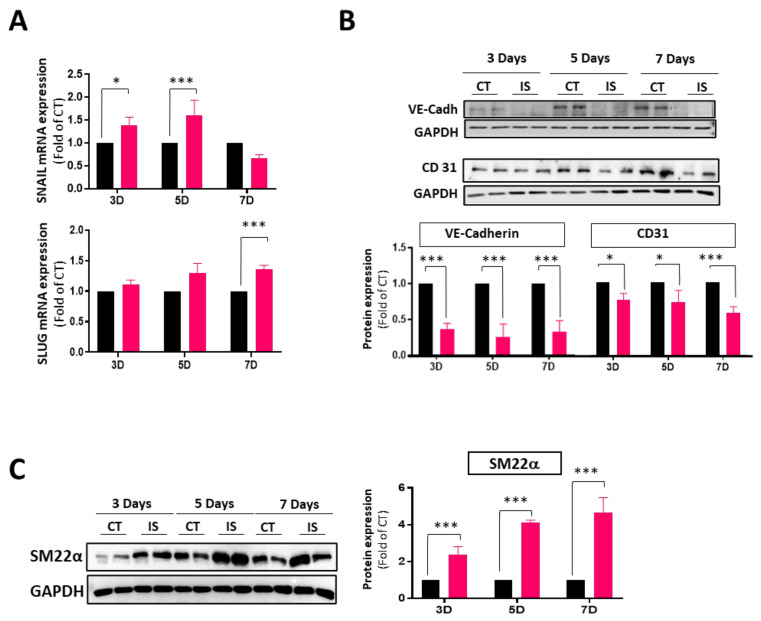
IS induces endMT in hVECs. Cells were exposed to IS 250 μM or PBS for 3, 5, and 7 days. Subsequently, cells were collected for analyses of mRNA and protein expression. (**A**) RT-qPCR of CT- and IS-treated hVEC extracts showing mRNA expression of *Snail and Slug*. (* *p* < 0.01; *** *p* < 0.0001; *n* = 3); (**B**) Western blot analysis and quantification of endothelial markers (VE-Cad and CD31) in hVECs. Experiments were performed in duplicate (* *p* < 0.01; *** *p* < 0.001; *n* = 3); (**C**) Immunoblot analysis and quantification of mesenchymal marker SM22α in hVECs (*** *p* < 0.001; *n* = 3). Black bar (CT) and red bars (IS). Experiments were performed in duplicate.

We and others have demonstrated that VECs contribute to calcific aortic valve disease by undergoing EndMT, a process marked by the loss of endothelial cell markers and the expression of myofibroblast cell markers [12,31,32]. Therefore, we studied whether IS may induce EndMT. Figure 2 shows that VEC exposure to IS for 3–7 days induced the early mRNA expression of Snail and Slug, two transcription factors involved in EndMT (Figure 2A). Indeed, IS induced the progressive loss of endothelial cell markers CD31 and VE-Cadherin (Figure 2B), while inducing the expression of mesenchymal markers such as SM22α (Figure 2C). 

### 3.3. IS Effects on VEC Transdifferentiation Are Mediated by Decreased ILK Expression

**Figure 3:**
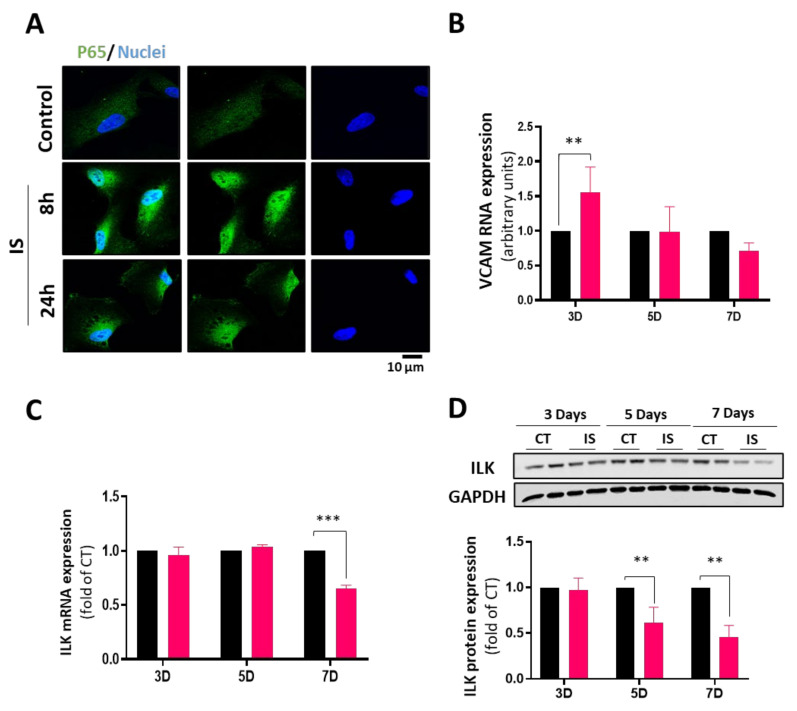
Indoxyl sulfate induces inflammation and a decrease in ILK levels in hVECs. hVECs were treated with IS (250 μM) (**A**) Immunofluorescence representation of NF-kB (green) translocation to nuclei (Hoechst blue) in cells treated with IS for 8 and 24 h; (**B**) RT-qPCR of CT- and IS-treated hVEC RNA extracts showing mRNA expression of VCAM-1 (** *p* < 0.001; *n* = 3); (**C**) RT-qPCR of CT- and IS-treated hVEC RNA extracts showing mRNA expression of ILK (*** *p* < 0.0001; *n* = 3); (**D**) Western blot analysis and quantification of ILK in hVECs treated with IS or CT for 3–7 days (** *p* < 0.001; *n* = 3). Black bar (CT) and red bars (IS). Experiments were performed in duplicate.

We first tested the inflammatory pathway as an early event in the calcific process to explore the molecular pathways leading to IS-induced VEC osteoblastic transformation. IS has been shown to promote endothelial inflammation by an NF-κB pathway [33,34]. We explored the NF-κB nuclear translocation by detecting the p65 subunit and VCAM-1 gene expression as a surrogate of NF-κB-dependent gene expression. Figure 3A shows that NF-κB translocates to the nucleus eight hours after VEC exposure to IS. VCAM-1 mRNA expression was induced three days after IS treatment, indicating the activation of this inflammatory pathway (Figure 3B). 

We have previously demonstrated a link between decreased ILK expression in valve endothelial cells and aortic valve calcification in humans [12]. In addition, ILK expression levels are regulated by inflammation [35]. As shown in Figure 3C,D, ILK expression progressively decreased in VECs in response to IS treatment at the mRNA and protein levels. These results strongly suggest a link between IS, ILK decreased expression, and calcification. 

**Figure 4:**
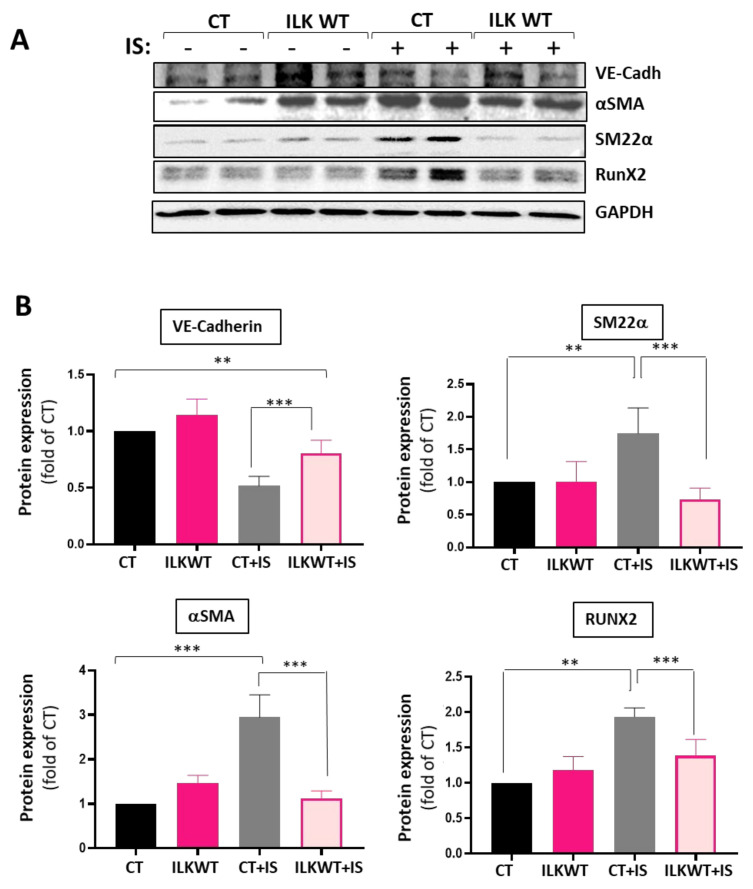
ILK overexpression in VECs treated with IS reverses the pathological phenotype. hVECs were treated and transfected with V5 tagged-ILK-WT (ILKWT) or empty plasmid (CT) and treated with IS 250 μM or PBS for seven days. (**A**) Representative Western blot and (**B**) quantification of EndMT markers and RUNX2 (** *p* < 0.001; *** *p* < 0.0001; *n* = 3). Experiments were performed in duplicate.

Decreased ILK levels in vascular endothelium induce endothelial dysfunction by uncoupling eNOS [36], and decreased levels of ILK are involved in aortic valve calcification [12]. To gain a deeper understanding of the role of ILK on the effects of IS, we overexpressed ILK in hVECs. VECs were transfected with a V5 tagged-ILK Wild-type (ILK WT) plasmid or empty plasmid as CT. As expected, ILK levels were increased in V5-ILKWT transfected cells, and a reduction was experienced in IS-treated cells transfected with the empty plasmid (Figure S1). Figure 4 shows that ILK overexpression prevented the IS-induced reduction in VE-Cadherin levels. Moreover, ILK overexpression prevented the transition to a mesenchymal phenotype since both αSMA and SM22α markers were reduced in ILK WT transfected cells exposed to IS compared with cells transfected with the empty plasmid. More importantly, ILK overexpression prevented RUNX2 overexpression induced by IS. 

**Figure 5:**
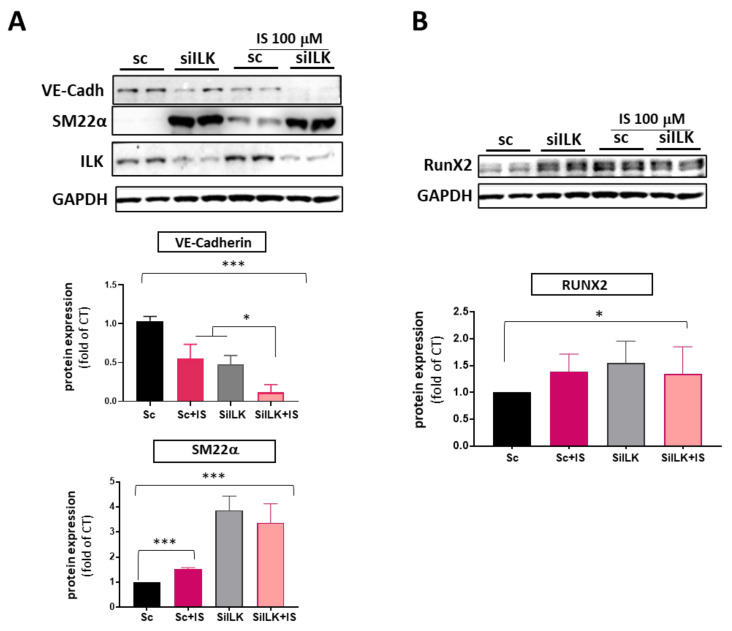
A lower dose of IS enhances EndMT induced by ILK silencing but does not potentiate increased RUNX2 levels. hVECs were transfected with siRNA targeting ILK and exposed to a lower dose of IS (100 μM) for 5 days. (**A**) Western blot analysis and quantification of endothelial (VE-Cadherin) and mesenchymal markers (SM22α) (* *p* < 0.01; *** *p* < 0.0001; *n* = 3); (**B**) Immunoblot analysis and quantification of RUNX2 (* *p* < 0.01; *n* = 3). Both experiments were performed in duplicate.

These results indicate that ILK plays a role in the pro-calcifying effects of indoxyl sulfate at a very high dose. Therefore, we decided to address whether a lower amount of IS could potentiate calcific changes in VECs with decreased ILK expression, a situation associated with endothelial dysfunction and the initiation of CAVD, which could help to explain the increased susceptibility to aortic valve calcification in patients with CKD. To investigate this, we exposed ILK-silenced VECs to a dose of IS 100 μM, equivalent to an earlier stage of CKD, which did not induce VEC toxicity. As shown in Figure 5A, ILK silencing induced EndMT events such as VE-Cadherin loss and SM22α increased expression. IS 100 μM treatment could not decrease ILK expression, but it decreased VE-Cadherin protein levels and induced a moderate increase in SM22α levels compared to VECs silenced with Scramble siRNA (SC). While IS treatment potentiated ILK silencing effects on VE-Cadherin levels, it did not further potentiate the increase in SM22α protein levels observed in ILK-silenced cells.

Regarding RUNX2, we observed a similar effect. IS 100 μM alone increased the expression of RUNX2, indicating a procalcific effect, but it did not potentiate the effects of ILK silencing (Figure 5B). Thus, even a moderate increase in circulating indoxyl sulfate levels can potentiate the effects of decreased ILK levels on the endothelial cell phenotype, which may promote endothelial cell barrier disruption and endothelial dysfunction, facilitating the calcific pathway.

## 4. Discussion

This study presents novel findings demonstrating that indoxyl sulfate induces endothelial damage in valvular endothelial cells, leading to endothelial-to-mesenchymal transition (EndMT) and osteogenic transformation. These effects are mediated, at least in part, through a mechanism that involves integrin-linked kinase (ILK).

Human Valvular Endothelial Cells exposed to 250 μM IS, a dose similar to analogous studies using different endothelial cell lines and associated with advanced CKD [23,27,28], induced a significant decrease in cellular viability of hVECs after a seven-day exposure. However, 100 μM IS did not show an evident toxic effect. As some authors have already demonstrated, this decrease in viability may be attributed to an imbalance in nitric oxide levels in favor of an increase in oxidative stress [17,22], indicating that IS induces endothelial dysfunction. Endothelial dysfunction is the earliest hallmark of CAVD, so the fact that IS induces reduced viability in VECs leads us to investigate the link between IS and CAVD in VECs.

Several studies have already confirmed that in the presence of IS the NF-κB pathway is activated in various types of cells, and we have observed that this also occurs in hVECs [15,37,38]. The activation of NF-κB in VECs indicates an active inflammatory process, confirmed by the increase in VCAM-1 mRNA in the first 72 h. Moreover, as other authors have observed, the NF-κB pathway induces the activation of RUNX2 [37,39], and in our case, we observed an increase at five days. The RUNX2 pathway is highly active in late-stage CAVD as it triggers the transition of valve cells (mainly VICs, but also transdifferentiated VECs) into an osteoblastic phenotype [39]. For this reason, the significant increase in RUNX2 expression on the fifth day of indoxyl treatment and the increased calcification observed in IS-exposed VECs is particularly noteworthy since it confirms that this pathway is also activated in this cell type. 

It is accepted that the endothelial phenotype switch by EndMT contributes to calcification [31,40]. In a pathological situation, VECs could undergo dysregulated EndMT and further differentiate in an osteogenic phenotype, contributing to the progression of CAVD. As previously observed with other types of endothelial cells, VE-Cadherin drastically decreases its expression [19,41] in response to IS, as evidenced by both qPCR and immunoblot analyses. VE-Cadherin decreased expression and reduced CD31 levels may indicate a pronounced loss of the endothelial phenotype in hVECs induced by IS. 

Colombo et al. reported a significant increase in epithelial–mesenchymal transition (EMT) markers in their study with microvascular endothelial cells in the context of exposure to IS alone, as opposed to a combination of uremic toxins [15]. While they did not explore the underlying reasons and mechanisms, they did encourage further investigation into whether exposure to IS could induce the Endothelial-to-Mesenchymal Transition (EndMT) process. Our study observed that SNAIL and SLUG, transcription factors that can trigger EndMT [11,42,43], increase significantly as early as three days, maintaining elevated expression until the seventh day. Furthermore, a notable elevation in SM22α and αSMA was observed within 72 h. Other studies also observed an elevation of αSMA in response to an equivalent dose of indoxyl sulfate in tubular cells, concluding that this uremic toxin is closely associated with an active cellular transdifferentiation process [44]. 

However, the most surprising finding was that ILK protein decreased between five and seven days of IS treatment. Our previous studies demonstrated that the decline in ILK is closely associated with increased RUNX2 in hVECs, leading to calcification by activating the TGFβ pathway [12]. Upon examining ILK mRNA expression, a notable decline was observed on the seventh day, while no differences were evident on day five. This discrepancy does not align with the protein-level findings and may indicate an IS-induced reduction in ILK mRNA stability. Another possibility is that inflammatory changes induced by IS are behind the reduced ILK levels. As our group described before, ILK levels can be regulated by inflammation, triggering its degradation in the lysosomes [35]. Since indoxyl sulfate induces VEC inflammation, more studies will be necessary to determine whether this could be the mechanism behind decreased ILK levels. Nevertheless, the ILK overexpression experiments demonstrated that ILK prevents endothelial-to-mesenchymal transition and RUNX2 expression induced by IS, although it only partially reverses the loss of the endothelial cell markers. Therefore, augmenting ILK levels mitigates the endothelial cell transition to a mesenchymal and pro-calcific phenotype, pointing clearly to ILK as a critical mediator of IS effects in VECs.

Next, we exposed ILK-silenced VECs to a lower dose of IS, equivalent to an earlier step of CKD. The results obtained from this experiment indicated that IS potentiated the loss of the endothelial phenotype in the endothelium that already had lower ILK levels. This effect may be attributed to the fact that both the presence of IS and the absence of ILK contribute to the decrease in nitric oxide, thereby intensifying the loss of Ve-Cadherin [10,36]. In contrast, the increase in SM22α expression induced by IS did not differ from the effect of ILK silencing, nor did the expression of RUNX2 increase with 100 μM of IS. As a result, we concluded that IS at this dosage did not potentiate the effects of ILK silencing to complete the EndMT and proceed to the osteoblastic transformation of VECs. Our results suggest that a moderate increase in circulating IS could synergistically impair valvular endothelial dysfunction and barrier function, paving the way for the inflammatory changes that induce CAVD. However, it may be possible that IS cannot potentiate further the osteoblastic transformation of ECs in cells expressing very low ILK levels. Therefore, it would be necessary to carry out experiments involving a less pronounced silencing of ILK to understand accurately the reason behind this effect. 

These two results also suggest that some of the indoxyl sulfate effects reported may be ILK-independent. Several reports indicate that the blockade of the IS-aryl hydrocarbon receptor (AhR) signaling pathway may protect endothelial cells from ischemic insult and reduce endothelial dysfunction [45]. In addition, IS activates AhR and the NF-κB pathway in valve interstitial cells, which induces IL-6 promoting VIC mineralization [37]. Thus, a more detailed exploration of this pathway is also necessary to completely elucidate the contribution of VECs to the mineralizing effects of IS in endothelial cells. 

## 5. Conclusions

Our findings demonstrate that uremic toxins, particularly indoxyl sulfate (IS), play a critical role in CAVD pathogenesis by causing valve endothelium to undergo EndMT and calcification. Our results strongly suggest that ILK is essential in the pro-calcifying effects induced by indoxyl sulfate. Preserving ILK levels in valve endothelium can offer new insights into managing CAVD in CKD patients.

## Data Availability

Data are contained within the article and Supplementary Materials.

## Supplementary Materials

The following supporting information can be downloaded at: https://www.mdpi.com/article/10.3390/cells13060481/s1, Table S1: Reagents and Antibodies; Table S2: Primers, Figure S1: ILK overexpression in VECs treated with IS.
