# Supplementary material for: Indoxyl Sulfate-Induced Valve Endothelial Cell Endothelial-to-Mesenchymal Transition and Calcification in an Integrin-Linked Kinase-Dependent Manner
Source: Cells. 2024 Mar 8;13(6):481. doi: 10.3390/cells13060481 (PMC10969166; doi:10.3390/cells13060481)
Supplement: Supplementary file 1 [file cells-13-00481-s001.zip › cells-2828676-supplementary.pdf]

Table S1. Reagents and Antibodies. Cell culture materials were procured from Lonza (located in Basel, Switzerland). Fetal bovine serum was sourced from Gibco (headquartered in Waltham, MA, USA), while matrigel was obtained from Corning (based in Corning, NY, USA). Sigma Aldrich (based in San Luis, MO, USA) supplied cell culture-grade gelatin, Alizarin Red, beta-glycerolphosphaten and ascorbic acid. Dispase II was acquired from Roche (Switzerland), and Fluorsave Reagent was obtained from Merck (based in Darmstadt, Germany). A detailed list of the antibodies utilized throughout the study is presented below:

Antibody	Supplier	Reference	Dilution
Anti-ILK	RD Systems	MAB374	WB: 1-1000
Anti-VE-cadherin	Santa Cruz	sc-9989	WB: 1-250
Anti-CD31	Abcam	ab32457	WB: 1-500
Anti-RunX2	Santa Cruz	Sc-39031	WB: 1-500
Anti-αSMA	Thermo Scientific	MA1-06110	WB: 1-1000
Anti-V5	Thermo Scientific	R960-25	WB: 1-500
Anti-Transgelin	Santa Cruz	Sc-53932	WB: 1-1000
Anti-Osteocalcin	Santa Cruz	sc-365797	WB: 1-500
Anti-p65	Santa cruz	sc-372-R	IF: 1-250
HRP-conjugated anti-Mouse secondary antibody	Invitrogen	A16072	WB: 1-10000
HRP-conjugated anti-Rabbit secondary antibody	Invitrogen	31466	WB: 1-5000
Alexa fluor 488 conjugated Goat anti- Rabbit secondary antibody	Abcam	ab150081	IF: 1-500

Table S2. Primers The primers employed in the present study are supplied by Sigma-Aldrich (St. Louis, MO, USA) are as follows:

GENE	Forward Sequence (5´-3´)	Reverse Sequence (5´-3´)
RunX2	GGCGGGTAACGATGAAAATT	GAGGCGGTCAGAGAACAAACTA
ALP	GCTGTAAGGACATCGCCTACCA	CCTGGCTTTCTCGTCACTCTCA
Snail	GGCAATTTAACAATGTCTGAAAAGG	GAATAGTTCTGGGAGACACATCG
Slug	ACTCCGAAGCCAAATGACAA	CTCTCTCTGTGGGTGTGTGT
VCAM-1	GTGGACATAAGAAACTGGAAAAGGG	CATTCACGAGGCCACCACTC
ILK	GTCTTGCAAACCCGTCTCTGCG	CAGAGGTGTCAGTGCTGGATG
β-Actin	CTTAGTTGCGTTACACCCTTTCTTG	CTGTCACCTTCACCGTTCCAGTTT

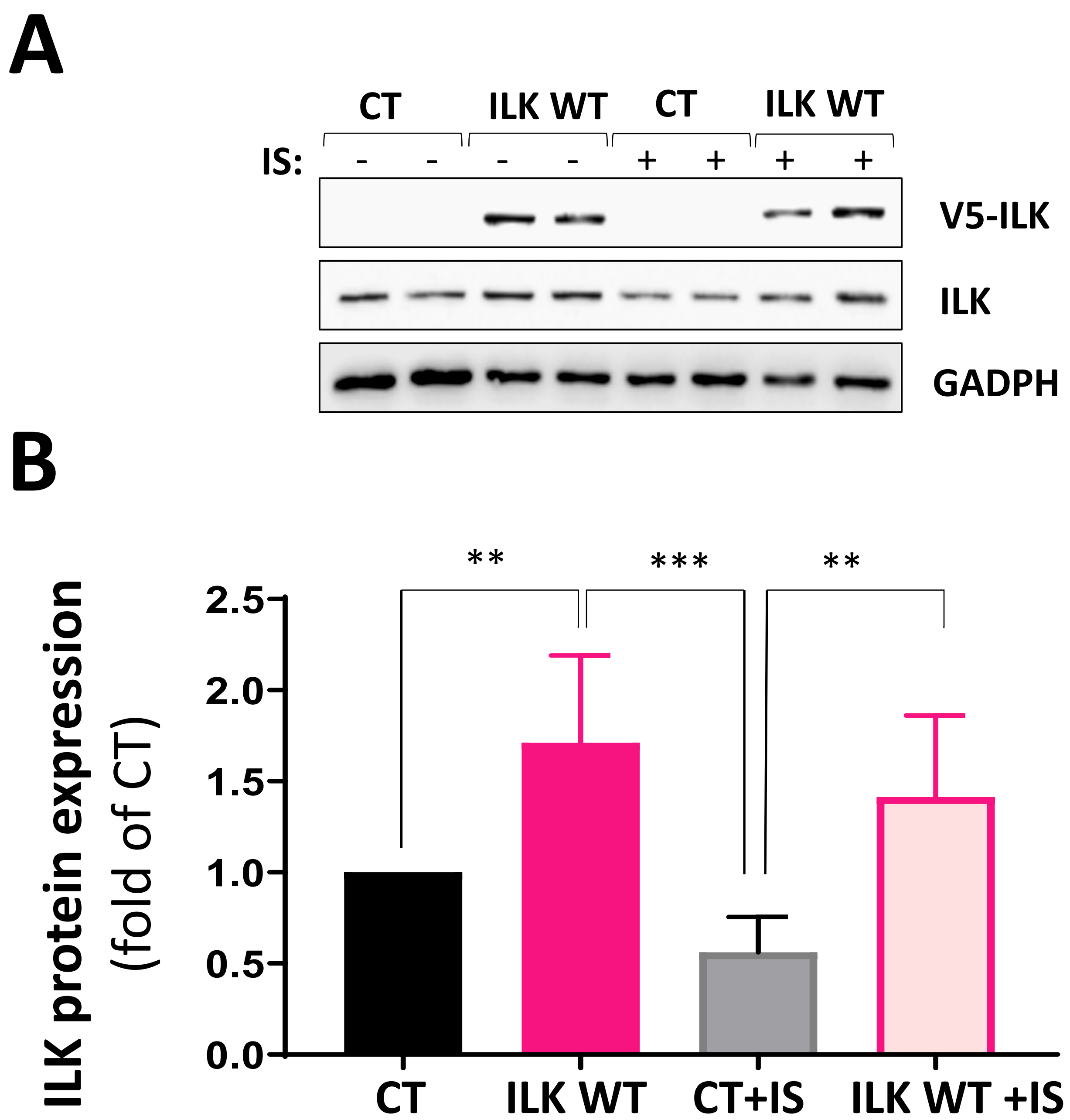

Supplementary Figure S1. ILK overexpression in VECs treated with IS. hVECs were transfected with V5-tagged ILK-WT(ILK WT) or empty plasmid (CT) and treated with IS 250 μM or PBS for seven days. **A.** Western blot analysis of V5-ILK and ILK. GADPH expression was used as loading control, **B.** Quantification of ILK levels expressed as fold of CT (** p< 0.001; ***p<0,0001; n = 3) Experiments were performed in duplicate.
